# Supplementary material for: Content-rich biological network constructed by mining PubMed abstracts
Source: BMC Bioinformatics. 2004 Oct 8;5:147. doi: 10.1186/1471-2105-5-147 (PMC528731; doi:10.1186/1471-2105-5-147)
Supplement: Additional File 2 — The original results of the above study (non-essential files are deleted to keep the file size under the limit set by BMC bioinformatics). [file 1471-2105-5-147-S2.bz2 › chilibotAdditionalFile2/dip20/100ID7759493E427/html/P_NP.html]

 


 **P** and **NP** 
  
Found 2734 abstracts in PubMed, retrieved 20.  
 

 What does Google say? 
 PDF only 
| .edu only 

---

**Interactive relationship** (e.g. stimulation, inhibition, etc)

**Stimulatory relationship**- Pregnant and  **NP**  ileal energy use increased linearly  **P**  <.  Ref: 12854824 J Anim Sci, 2003
- both PR and  **NP**  decreased with time, but  **NP**  increased on d 270  **P**  = 0.09.  Ref: 12854824 J Anim Sci, 2003
**Neutral relationship**- Liver protein concentration was less  **P**  = 0.07 in PR than in  **NP**  heifers  **NP**  = 291.1 vs. PR = 210.5 33.9 mg g.  Ref: 12854824 J Anim Sci, 2003
- The independent RFs of  **NP**  were a history of  **NP**  during the previous 6 months OR = 4.50 and oxygen therapy OR = 16.15,  **P**  < ;.  Ref: 12834521 J Am Geriatr Soc, 2003
- **P**  rats were found to have significantly greater brain damage, compared with  **NP**  rats, in the posterior perirhinal and posterior entorhinal cortexes, 239% 50%  **p**  <.  Ref: 12878913 Alcohol Clin Exp Res, 2003
- Phospho extracellular signal regulated kinase immunohistochemistry stained prominently in damaged brain areas.CONCLUSIONS The  **P**  rat line, a genetic model of alcoholism, shows greater region specific brain damage due to binge ethanol treatment than its genetic counterpart, the  **NP**  rat line.  Ref: 12878913 Alcohol Clin Exp Res, 2003
- Enzymatic transglycosylation using four possible monodeoxy analogs of  **p**  nitrophenyl alpha D glucopyranoside Glc alpha O pNP  [ **NP** ] , modified at the C 2, C 3, C 4, and C 6 positions 2D, 3D, 4D, and 6D Glc alpha O pNP  [ **NP** ] , respectively, as glycosyl donors and six equivalents of ethyl beta D thioglucopyranoside Glc beta S Et as a glycosyl acceptor, to yield the monodeoxy derivatives of glucooligosaccharides were done.  Ref: 12834279 Biosci Biotechnol Biochem, 2003
- The average exercise related decreases in UtBF  **P**  16 4% vs  **NP**  48 4% and UtC  **P**  27 4% vs  **NP**  54 4% were attenuated in the  **P**  rabbits.  Ref: 12869366 Am J Physiol Regul Integr Comp PhysiolAm J Physiol Regul Integr Comp Physiol, 2003

**Non-interactive relationship** (e.g. studied together, co-existance, homology, etc.)

- Although few systems have been studied in detail, F like T4SS appear to be involved in the transfer of DNA only whereas  **P**  and I type systems appear to transport protein or nucleoprotein  [ **NP** ]  complexes.  Ref: 12855161 FEMS Microbiol Lett, 2003
- To this aim, rats of the sNP and Sardinian alcohol preferring sP lines were exposed to a 108 day sucrose fading procedure, which was virtually identical to that previously used with  **NP**  and  **P**  rats.  Ref: 12878272 Alcohol, 2003
- Much greater vitellogenin compared to the background levels were induced in the male medaka by separate exposure to 100 microg L of  **p**   **NP** , 00 microg L of BPA and 0.05 microg L of E2.  Ref: 12830940 Water Sci Technol, 2003
- The relative potencies of these chemicals descended in the order of E2>> **p**   **NP** >BPA.  Ref: 12830940 Water Sci Technol, 2003
- snail, abalone, and limpet origins were investigated with assorted  **p**  nitrophenyl pNP  [ **NP** ]  di O sulfonated beta D galactopyranosides and beta lactosides SO 3 Gal, 6 SO 3 Lac, 6SO 3 Gal, 6 SO 3 Gal, 4 SO 3 Gal 5, and SO 3 GalNAc 6.  Ref: 12851934 Chembiochem, 2003
- Animals were killed, transcardially perfused, and fixed, and their brains were removed, sectioned, and stained by using the amino cupric silver stain of de Olmos or by using immunohistochemistry for phospho extracellular signal regulated kinases and other antigens.RESULTS Significant brain damage was found in the olfactory bulbs, posterior perirhinal cortex, and entorhinal cortex in both  **P**  and  **NP**  rats.  Ref: 12878913 Alcohol Clin Exp Res, 2003
- Pregnancy did not affect the average steady state renal SNA response expressed relative to maximum activity  **P**  24 1% vs  **NP**  23 2% of maximum smoke elicited activity and increased the average renal SNA response expressed relative to resting activity  **P**  155 19% vs  **NP**  84 23% from rest,  **P** =0.03 At rest, UtBF  **P**  13 3 vs  **NP**  1.9 0.3 ml min and UtC  **P**  22 5 vs  **NP**  2.8 0.5 mmHg ml.  Ref: 12869366 Am J Physiol Regul Integr Comp PhysiolAm J Physiol Regul Integr Comp Physiol, 2003
- The uterine artery blood flow and renal sympathetic nerve activity SNA responses to treadmill exercise were evaluated in 12 nonpregnant  **NP**  and 17 term pregnant  **P**  rabbits.  Ref: 12869366 Am J Physiol Regul Integr Comp PhysiolAm J Physiol Regul Integr Comp Physiol, 2003
- Covalent attachment of 2C5 antibody was performed via a micelle incorporated PEG PE with the distal terminus of the PEG block activated with  **p**  nitrophenylcarbonyl group pNP  [ **NP** ]  PEG PE.  Ref: 12881194 J Drug Target, 2003
- Binge Ethanol Treatment Causes Greater Brain Damage in Alcohol Preferring  **P**  Rats Than in Alcohol Nonpreferring  **NP**  Rats.  Ref: 12878913 Alcohol Clin Exp Res, 2003
- Mature male medaka were continually exposed to four chemicals,  **p**  n nonylphenol  **p**  n  **NP** , nonylphenol  **p**   **NP** , bisphenol A BPA and 17beta estradiol E2 to evaluate their estrogenic activities in the laboratory.  Ref: 12830940 Water Sci Technol, 2003
- Parts per million and parts per billion concentrations of these species were found in the air passing through the tubing the plasticizers di 2 ethylhexyl phthalate DEHP and di ethyl phthalate DEP, the antioxidants butylated hydroxy toluene BHT and  **p**  nonylphenol  **p**   **NP** , and the contaminant from commercial preparation of DEHP 2 ethylhexanol 2 EH.  Ref: 12833390 Biomed Chromatogr, 2003
- DeltaFENa was 7.22 1.03% for  **NP**  VE, 9.89 1.85% for  **NP**  L NMMA, and 17.66 1.85% for  **NP**  VE L NMMA  **P** < ; 0.05 vs.  **NP**  VE and  **NP**  L NMMA.  Ref: 12876067 Am J Physiol Renal PhysiolAm J Physiol Renal Physiol, 2003
- 0.01, but ileal energy use by PR was less throughout gestation  **P**  = 0.07 than ileal energy use by  **NP** .  Ref: 12854824 J Anim Sci, 2003
- To investigate the role of genetics in binge drinking induced brain damage, we studied bidirectionally selected rat lines, the alcohol preferring  **P**  and the alcohol nonpreferring  **NP**  rat lines.METHOD  **P**  and  **NP**  rats were treated with a 4 day binge ethanol protocol.  Ref: 12878913 Alcohol Clin Exp Res, 2003
